# Correlation between PET-CT and ct in the staging after the treatment of head and neck squamous cell carcinoma

**DOI:** 10.1016/j.bjorl.2021.11.008

**Published:** 2021-12-07

**Authors:** Fernando García-Curdi, Yolanda Lois-Ortega, Ana Muniesa-del Campo, Amaranta McGee-Laso, José Miguel Sebastián-Cortés, Héctor Vallés-Varela, Julio José Lambea-Sorrosal

**Affiliations:** a Hospital Santa Bárbara, Department of Otorhinolaryngology, Soria, Spain; b Universidad de Zaragoza, Faculty of Veterinary Sciences, Department of Animal Pathology, Zaragoza, Spain; c Hospital Ramon y Cajal, Department of Preventive Medicine and Public Health, Madrid, Spain; d Hospital Clínico Universitario Lozano Blesa, Department of Otorhinolaryngology, Zaragoza, Spain

**Keywords:** Head and neck squamous cell carcinoma, Restaging, Correlation, Positron emission computed tomography, Computed tomography

## Abstract

**Objective:**

The aim of this study is to find out if a single imaging test is enough to follow-up on an oncological post-treatment patient. In such a case, we would know which was more valuable after comparing the two, by CT or PET-CT.

**Methods:**

Between January 2012 and July 2018, we collected data from all patients with previous medical history who were treated with a head and neck squamous cell carcinoma in our hospital, through surgery or by using an organ preservation protocol which we had done. Patients were required to have a CT and a PET-CT performed in a maximum period of 30 days between techniques. We compared the post post-treatment stage given to each case by using only the physical examination (only the CT and the PET-CT), with the ones given by the Tumor Board. After treatment, we analysed the similarity through Cramer’s V statistic test.

**Results:**

We performed a comparative analysis, obtaining a correlation of 0.426 between the stages given by the Tumor Board and the one assigned based on physical examination, without imaging techniques. By only using the computed tomography as an imaging method the correlation was 0.565, whereas with only the use of positron emission computed technology, it was estimated at 0.858. When we compared the statistical association between stages using exclusively one of the two imaging techniques, the correlation was 0.451.

**Conclusion:**

Independent of the modality, we have demonstrated that in patients who have received previous treatment, there was a higher correlation in the stages with respect to the diagnostic method conducted by the Tumor Board using PET-CT as the sole image.

**Level of evidence:**

Level 1.

## Highlights

• Head and neck cancer recurs more frequently in the first 2–3 years after treatment.
• Early detection of recurrence events is important.
• Diagnostic imaging methods are an indispensable tool in the follow up of the disease.
• The post-treatment neck study is controversial.

## Introduction

Malignant neoplasms of the head and neck recur more frequently in the first 2–3 years after treatment. The most common sites of relapse are located in the primary tumor and in the regional lymph nodes. Any new soft tissue injury arising in or around the treated site would make us suspect that it is a recurrence.^1^, ^2^

Early detection of recurring situations is important. Thus, diagnostic imaging methods are indispensable in the follow up of the disease. One of these techniques is the Positron Emission Tomography/Computed Tomography or PET-CT. Patients with negative post-treatment PET-CT develop recurrences of the disease less frequently, and those with a positive after treatment PET-CT allows for the early detection of non-remissions and recurrences. These would make it possible for the surgical rescue of patients with operable disease. Or the start of a systemic treatment that can improve the long-term prognosis in those with non-operable disease.^3^, ^4^

Post-treatment neck study is controversial, as there are discrepancies on what imaging technique is to be used. The National Comprehensive Cancer Guidelines Squamous Cell Carcinoma of the Head and Neck Network (NCCN) recommends performing PET-CT after radiotherapy or chemoradiotherapy, to evaluate the need for cervical lymph node dissection.^5^ The debate on the necessity to perform post-treatment cervical dissection in patients who at diagnosis had extensive cervical lymph node metastases (N2 or N3 disease) was studied through a large, randomized trial. The study showed that surveillance PET-CT did not decrease survival, even after having fewer surgical interventions and was more cost-effective compared to prophylactic castings.^6^

There is limited scientific evidence regarding the comparison of CT and PET-CT to determine tumor extension. Of special mention was the article that analysed the existing correlation among staging tumors that had not received treatment previously, using only a single imaging method, or using two imaging techniques (CT and PET-CT), simultaneously in patients diagnosed with SCCC.^7^ In this article, a higher precision of PET-CT at the time in detecting cervical lymph metastasis and distant ganglion cells was observed.

In this work, an analysis of the same parameters was carried out to check if the correlation was maintained, in patients who had been previously treated through chemotherapy, radiation therapy, surgery, or a combination of these. At the end of the treatment, all patients were re-evaluated by the Tumor Board, after having the CT and PET-CT. So, a new stage was released, we call T, N and M or (T = Primary tumor), (N = Pathological adenopathy) and (M = distant metastases) which was given post-treatment. The new stage given by the Tumor Board of our Hospital, based on all available imaging tests (CT and PET-CT), was compared with that given by using only physical examination, or exclusively using CT or PET-CT.

The objective of this study is to check whether it is sufficient to make a follow-up of the post-treatment cancer patient performing exclusively a single image. And in such a case, which of the two we compare (CT or PET-CT) has a higher performance or whether the patient can benefit from performing both tests.

## Methods

Data from 125 patients were collected from January 2012 to July 2018, under the approval of the Research Ethics Committee of the Region of Aragón (CEICA) (C.P. – C.I. PI18/061 – Acta nº 06/2018) and by the direction of Hospital Clínico Universitario Lozano Blesa in Zaragoza.

The inclusion criteria were having suffered from SCCC in any location (from sinuses, nasopharynx, oropharynx, hypopharynx or larynx), at any stage, and having previously received treatment, either by surgery or by organ conservation protocol. Once the treatment is finalised, patients must have a post-treatment CT and PET-CT performed with a maximum period of a 30-day interval between the two tests. Stages were assigned according to the American Joint Classification Committee on Cancer Staging Manual (AJCC) (7th edition). Patients who do not meet these requirements were excluded from the study.

This is a retrospective observational study carried out at the only third class-level center. All patients at the end of treatment were reassessed through a physical examination (consisting of direct vision, palpation, and flexible laryngoscopy), a cervical-thoracic CT contrast and a PET-CT.

The protocol of the imaging tests (CT and PET-CT) was as follows:

CT scan (of the neck and chest) was performed after an intravenous iodine contrast injection. The cervical study focused on the head and neck region (from the base of the skull to the thoracic inlet with a slice thickness of 1 mm). The thoracic study was performed from the thoracic inlet to the adrenal glands (3 mm slice thickness).

PET-CT was performed in patients with at least 6 h of fasting. Sixty minutes before the test, they were given an intravenous injection of Fluorodeoxyglucose (FDG) using a 3–4 MBq/kg dose. The images were acquired from the head to the mid-thighs in the axial plane. Then they were reconstructed in coronal and sagittal planes.

Once the patients finalised the therapy, all cases were again presented to the Multidisciplinary Tumor Board of Hospital Clínico Universitario Lozano Blesa to evaluate the response to treatment. This Board is formed by the Otolaryngologist, Oncologists, Radiotherapists, Pathologists and Radiologists. After the presentation of every case, including the physical examination (performed by the Otolaryngologist) and diagnostic tests (evaluated by radiologists and specialists in nuclear medicine, depending on whether it is CT or PET-CT respectively) a post-treatment TNM stage of the tumor is established according to the AJCC classification.

After receiving treatment, all patients were reassessed. It was considered a tumor recurrence and there was no tumor persistence, when the lesions completely disappeared within 3 months. A comparison of the stages given to each case was based solely on the physical examination (only in CT and only in PET-CT), regarding the post-treatment stages given by the Tumor Board. We analysed the compatibility through Cramer’s V statistic test. In addition, we performed individualized correlation analysis between these methods: diagnoses of T (primary tumor), N (pathological adenopathies) and M (distant metastases) after the end of the primary treatment individually, by grouping tumors from all locations.

Data was analysed using IBM SPSS 19.0 for Windows (IBM Corp., Armonk, NY, USA). The association between the different techniques was established using the likelihood ratio statistical test and was quantified using Cramer’s V. The alpha error was set at 0.05 – which was equivalent to a 95% confidence level.

## Results

A total of 125 cases have been analysed, mainly on men (88.0%), with an age average of 61 years. The sample studied is described below (Table 1).

**Table 1:** Patients characteristics.

Characteristics	Number of patients
Sex	
Men	110 (88.0%)
Women	15 (12.0%)
Age	
Median age	61.08 (min. 34.71–max. 82.42)
Location of the primary tumour	
Paranasal sinuses	4 (3.2%)
Nasopharynx	16 (12.8%)
Oropharynx	31 (24.8%)
Hipopharynx	9 (7.2%)
Larynx	65 (52.0%)
Supraglottis	56 (44.8%)
Glottis	9 (7.2%)
Total	125 (100.0%)
Stage	
0	65 (52.0%)
I	1 (0.8%)
II	4 (3.2%)
III	8 (6.4%)
IVa	27 (21.6%)
IVb	o (0.0%)
IVc	20 (16.0%)

First, the correlation between the physical examination without the use of the imaging method was studied in relation to the T, N and M post-treatment stage given by the Tumor Board, using both imaging tests (PET-CT and TC). It should be noted that in 5 cases the physical examination was not included in the history of the patient, so that they were eliminated from this analysis. In our study it is observed that there was a very low correlation between the post-treatment stage awarded by the Board on Tumors and that obtained through physical examination, coinciding at 59.16% of the cases, producing an underestimation of 32.5% in our sample and overestimation at 8.33% of the cases analysed (Table 2). We see that there was a low correlation (Cramer’s V 0.426 statistically significant, *p* < 0.001) with the tendency to underestimate the tumor stage, when staging tumors without the use of imaging techniques. When evaluating the primary tumor, we observed a statistically significant correlation of 0.473 (*p* < 0.001), coinciding with 82.5% of the occasions, while under staging occurs in 12.5% and over staging in 13.33% of the cases (Table 2). When conducting the analysis of the locoregional lymph nodes affected, the correlation between staging based on palpation only and palpation-based staging in conjunction with PET-CT and CT at 0.514, which was statistically significant (*p* < 0.001); observing a match at 78.33% of the occasions between the detection of lymphadenopathy in consultation and their detection using both diagnostic imaging methods, producing an underestimation at 18.33% of the sample, and an overestimation only in 3 cases (Table 2). In the case of distant metastases, it was impossible to detect them without imaging tests, so their analysis was not very relevant.

**Table 2:** Correlation between the restaging obtained through physical examination and the one given by the Tumor Board.

	Tumor Board Stage								
**Physical Examination + CT Stage**		**0**	**I**	**II**	**III**	**IVa**	**IVb**	**IVc**	**TotaL**
	0	55	0	1	3	8	0	11	78
	I	2	1	0	0	0	0	1	4
	II	3	0	2	2	0	0	0	7
	III	3	0	1	3	6	0	2	15
	IVa	0	0	0	0	9	0	5	14
	IVb	0	0	0	0	1	0	0	1
	IVc	0	0	0	0	0	0	1	1
	Total	63	1	4	8	24	0	20	120
	Cramer’s V 0.426 *p* < 0.001								

	Tumor Board T						
**Physical Examination + CT T**		**There is no tumor**	**T1**	**T2**	**T3**	**T4**	**Total**
	There is no tumor	88	0	3	4	2	97
	T1	2	1	0	1	1	5
	T2	3	0	2	2	0	7
	T3	1	0	0	7	2	10
	T4	0	0	0	0	1	1
	TOTAL	94	1	5	14	6	120
	Cramer’s V 0.473 *p* < 0.001						

	Tumor Board N							
**Physical Examination + CT N**		**N0**	**N1**	**N2a**	**N2b**	**N2c**	**N3**	**Total**
	N0	83	4	1	5	5	0	98
	N1	2	3	1	1	1	0	8
	N2a	0	0	4	1	3	0	8
	N2b	0	0	0	3	1	0	4
	N2c	0	0	0	0	1	0	1
	N3	0	0	1	0	0	0	1
	Total	85	7	7	10	11		120
	Cramer’s V 0.514 *p* < 0.001							

When examining the correlation between the stages, after the end of the treatment, obtained through physical examination and CT compared to that given by the Tumor Board, we obtained a correlation of 0.565. There was a coincidence of 62.4% in the cases analysed, with an overestimation of 20.89% and an underestimation of 15.8% in the sample (Table 3). With respect to the compatibility, in determining the extension and size of the primary tumor once the treatment was over, we obtained a correlation of 0.502. By using CT as the only diagnostic imaging method, we obtained a coincidence of 73.6% of the cases, an over staging of 20.0% and under staging of 6.4% on the patients analysed (Table 3). When we studied the post-therapy locoregional lymph node involvement, there was a correlation of 0.531, with the CT coinciding with the Tumor Board in 79.2% of the cases, with an underestimation of 12.8% and an overestimation of 8.0% (Table 3). According to the detection of lymph node metastases at a distance post-treatment, a 0.673 agreement, with a 92% agreement, 7.2% under staging and an over staging of 0.8% (Table 3). When analysing the detection of metastases to distance, we obtain a compatibility of 0.673, coinciding in the diagnosis with the Tumor Board in 88.0% of the cases.

**Table 3:** Correlation between the restaging obtained by physical examination and CT, and the one given by the Tumor Board.

	Tumor Board Stage								
**Physical Examination + CT Stage**		**0**	**I**	**II**	**III**	**IVa**	**IVb**	**IVc**	**TotaL**
	0	39	0	1	1	4	0	3	48
	I	1	1	0	0	0	0	0	2
	II	8	0	3	0	2	0	1	14
	III	9	0	0	7	4	0	1	21
	IVa	7	0	0	0	17	0	4	28
	IVb	0	0	0	0	0	0	0	0
	IVc	1	0	0	0	0	0	11	12
	Total	65	1	4	8	27	0	20	125
	Cramer’s V 0.565 *p* < 0.001								

	Tumor Board T						
**Physical Examination + CT T**		**There is no tumor**	**T1**	**T2**	**T3**	**T4**	**Total**
	There is no tumor	72	0	3	2	2	79
	T1	2	1	0	0	0	3
	T2	9	0	3	0	0	12
	T3	10	0	0	13	1	24
	T4	4	0	0	0	3	7
	TOTAL	97	1	6	15	6	125
	Cramer’s V 0.502 *p* < 0.001						

	Tumor Board N							
**Physical Examination + CT N**		**N0**	**N1**	**N2a**	**N2b**	**N2c**	**N3**	**Total**
	N0	79	2	3	3	3	0	90
	N1	5	5	1	0	1	0	12
	N2a	0	0	2	1	1	0	4
	N2b	1	0	1	5	1	0	8
	N2c	2	0	0	1	8	0	11
	N3	0	0	0	0	0	0	0
	Total	87	7	7	10	14	0	125
	Cramer’s V 0.531 *p* < 0.001							

	Tumor Board M			
**Physical Examination + CT M**		**M0**	**M1**	**Total**
	M0	104	9	113
	M1	1	11	12
	Total	105	20	125
	Cramer’s V 0.673 *p* < 0.001			

We have studied the correlation between the post-treatment restaging obtained through the use of PET-CT and the physical examination compared to the one granted by the Tumor Board. When analysing the tumor stages, we have obtained a compatibility of 0.858 (Table 4). We observe that there was a coincidence of 93.6% of the cases analysed, with an underestimation of 3.2% and an overestimation of the same percentage (3.2%). When we compared the persistence or recurrence of the primary tumor, we obtained a statistically significant agreement of 0.852, with a coincidence of 96% in the cases, under staging of 2.4% and overestimation of 1.6% (Table 4). When studying the existence of metastatic cervical lymphadenopathy residuals, we observed a correlation of 0.969, with coincidence of 98.4%. There was only an underestimation in 1 case and an overestimation in another (Table 4). The correlation observed when detecting distant metastases post-treatment was 0.913, with coincidence between the PET-CT and the Tumor Board at 96.8% of the cases, with errors in 3 cases, 2 due to over staging and 1 by sub staging (Table 4).

**Table 4:** Correlation between the restaging obtained by physical examination and PET-CT, and the one given by the Tumor Board.

	Tumor Board Stage								
**Physical Examination + PET-CT Stage**		**0**	**I**	**II**	**III**	**IVa**	**IVb**	**IVc**	**Total**
	0	63	0	1	0	2	0	0	66
	I	1	1	0	0	0	0	0	2
	II	0	0	3	0	0	0	0	3
	III	1	0	0	7	0	0	0	8
	IVa	0	0	0	0	24	0	1	25
	IVb	0	0	0	0	0	0	0	0
	IVc	0	0	0	1	1	0	19	21
	Total	65	1	4	8	27	0	20	125
	Cramer’s V 0.858 *p* < 0.001								

	Tumor Board T						
**Physycal examination + PET/CT T**		**There is no tumor**	**T1**	**T2**	**T3**	**T4**	**Total**
	There is no tumor	95	0	2	0	1	98
	T1	1	1	0	0	0	2
	T2	0	0	4	0	0	4
	T3	1	0	0	15	0	16
	T4	0	0	0	0	5	5
	Total	97	1	6	15	6	125
	Cramer’s V 0.852 *p* < 0.001						

	Tumor Board N							
**Physycal Examination + PET/CT N**		**N0**	**N1**	**N2a**	**N2b**	**N2c**	**N3**	**Total**
	N0	87	0	0	0	1	0	88
	N1	0	7	0	0	0	0	7
	N2a	0	0	7	0	0	0	7
	N2b	0	0	0	9	0	0	9
	N2c	0	0	0	1	13	0	14
	N3	0	0	0	0	0	0	0
	Total	87	7	7	10	14	0	125
	Cramer’s V 0.969 *p* < 0.001							

	Tumor Board M			
**Physycal Examination + PET/CT M**		**M0**	**M1**	**Total**
	M0	102	1	103
	M1	2	19	21
	Total	104	20	124
	Cramer’s V 0.913 *p* < 0.001			

In our study, we have analysed the correlation in restaging using both imaging tests separately, that is, comparing the data obtained only by PET-CT and by CT. By emitting the tumor stage once after the treatment, we obtained a correlation of 0.451, with a coincidence of 56.0% of the cases analysed (Table 5). Regarding the analysis of persistence or recurrence of the primary tumor, the correlation obtained was 0.397, with a coincidence of 69.6% (Table 5). When analysing the cervical lymph nodes, we observed a compatibility of 0.535, with both diagnostic methods coinciding in 79.2% occasions (Table 5). In the study of the presence of distant post-treatment metastases, a compatibility of 0.579 was obtained, with a coincidence of 88.8% of the cases analysed.

**Table 5:** Correlation between the restaging obtained by the physical examination and PET-CT with respect to the stage obtained through physical examination and CT.

	Physical Examination + CT Stage								
**Physical Examination + PET-CT Stage**	**0**	**0**	**I**	**II**	**III**	**IVa**	**IVb**	**IVc**	**Total**
	0	37	1	9	9	9	0	1	66
	I	1	1	0	0	0	0	0	2
	II	1	0	2	0	0	0	0	3
	III	2	0	0	6	0	0	0	8
	IVa	4	0	2	4	14	0	1	2255
	IVb	0	0	0	0	0	0	0	0
	IVc	3	0	1	2	5	0	10	21
	Total	48	2	14	21	28	o	12	125
	Cramer’s V 0.451 *p* < 0.001								

	Physical Examination + CT T						
**Physical Examination + PET-CT T**		**There is no tumor**	**T1**	**T2**	**T3**	**T4**	**Total**
	There is no tumor	70	2	11	10	5	98
	T1	1	1	0	0	0	2
	T2	3	0	1	0	0	4
	T3	3	0	0	13	0	16
	T4	2	0	0	1	2	5
	Total	79	3	12	24	7	125
	Cramer’s V 0.397 *p* < 0.001						

	Physical examination + CT N							
**Physical Examination + PET-CT N**		**NO**	**N1**	**N2a**	**N2b**	**N2c**	**N3**	**Total**
	NO	79	5	1	1	2	0	88
	N1	2	5	0	0	0	0	7
	N2a	3	1	2	1	0	0	7
	N2b	2	0	1	5	1	0	9
	N2c	4	1	0	1	8	0	1144
	N3	0	0	0	0	0	0	0
	Total	90	12	4	8	11	0	125
	Cramer’s V 0.535 *p* < 0.001							

	Physical Examination + CT M			
**Physical Examination + PET-CT M**		**MO**	**M1**	**Total**
	MO	101	2	103
	M1	11	10	21
	Total	112	12	124
	Cramer’s V 0.579 *p* < 0.001			

## Discussion

We have observed the need to use imaging methods to assess the post-treatment response, and once radiotherapy is finished. There may be an inflammation of the region where the local tumor had settled, a hidden rest tumor or an underlying early recurrence.

In the same way, cervical palpation is less accurate in a treated neck, since the intense fibrosis of the tissues can mask deep lymphadenopathy, or by the simple fact that the lymph node metastases are of a size that does not allow Positive Predictive Value (PPV). There are studies that cipher sensibility, specificity and Negative Predictive Value (NPV) of a post-treatment physical examination in 73%, 45%, 41% and 77% cases respectively.^8^

Our study highlights the need for imaging tests performed on post-treatment tumor staging. We have made a comparison of the results of our study with respect to a previous study of our working group, in which the same parameters were analysed but in patients who had not received previous treatment,^7^ and we observed that the correlation decreases by not using image-based diagnostic techniques in patients already treated; the correlation decreased by assigning a tumor stage to each treated case, a decrease from 0.729 to 0.426 was observed, when the presence of the primary tumor drops from 0.878 to 0.473 and when detecting only regional lymph nodes by cervical palpation, the correlation decreased from 0.719 to 0.514, so that our analysis agreed with other published studies.^7^, ^8^

So far, CT is considered to be the standard imaging test for the diagnosis of CCC.^9^, ^10^ It has also been the most used test to study the response to treatment at both local and locoregional levels. However, as the post-treatment neck may be difficult to assess with image due to distortion of the anatomy and post radiation changes,^1^, ^2^ it is essential to obtain accurate images after treatment, especially in areas that are difficult to clinically evaluate, for the purpose of monitoring the patient.

CT is an imaging technique that can help differentiate recurrence of the post-treatment changes, since the use of PET-CT can generate false positives in the treated area, due to inflammatory changes.^11^ However, other studies have demonstrated the accuracy of PET-CT in the examination of patients with CCC post-treatment.^12^, ^13^, ^14^

In our study we observed that in patients who have received treatment, regardless of the modality, by physical examination and CT with respect to the Tumor Board, it decreased in relation to untreated patients. For this we have compared the results obtained in the present study with respect to those obtained in a previous one,^7^ where the same parameters were analysed but in previously untreated patients. The Greater discrepancies are more clearly observed when evaluating the extension of the post-treatment primary tumor, where the correlation drops from 0.912 to 0.502. It also decreases when performing a tumor re-staging in each case from 0.848 to 0.565, and in the detection of post-therapy pathological adenopathies from 0.742 to 0.531.

We have obtained a good correlation between the reestablishment granted by the Tumor Board and the re-stage given to each patient using PET-CT as the only one imaging method (at 0.858) compared to untreated patients (at 0.957). At the same time, we have obtained a correlation of 0.852 in the detection and re-staging of the primary tumor, with a low over staging and under staging, a very similar figure to the correlation obtained in untreated patients (at 0.960).

Numerous studies have analysed the usefulness of PET-CT in the detection of local tumor persistence or recurrence. Helsen et al. yielded figures on the sensitivity, specificity, PPV and NPV of PET-CT for the detection of local post-treatment residual in 91.1%, 87.0%, 77.3% and 95.3%, respectively.^15^ Therefore, negative findings on PET-CT will reduce the probability that there is residual disease after treatment to only 5%. When studying the time of relapse, it proved to be very effective in detecting recurrences that occur in the first 9 months after the test, with a sensitivity of 91.1%.

Regarding the detection of post-treatment pathological adenopathies, we have observed a correlation of 0.969, which indicates that PET-CT is quite an accurate diagnostic tool, since in the absence of other methods of imaging coincides with the Tumor Board in 98.4% of the cases. When comparing it with our study, it is striking that we found a better correlation in untreated patients (Cramer’s V 0.897).^7^

In a meta-analysis of 51 studies involving 2335 patients, the weighted average of sensitivity, specificity, PPV and NPV of PET-CT in the study of the post radiation cervical lymph nodes was 72.7%, 87.6%, 52.1% and 94.5%, respectively.^16^ Another study analysed the sensitivity, specificity, PPV and NPV values at 91%, 93%, 81%, 97% respectively. This means that the possibility of having a locoregional disease is 81% in the case of a positive PET-CT and 3% in the case of being a negative.^15^ Therefore, instead of performing a cervical emptying of all patients with tumors in advanced stages, it is possible to opt for observation in patients with negative post-treatment PET-CT and adenopathies smaller than 1 cm, due to the high NPV that this imaging test demonstrated.^5^

Another study by Sher et al. analysed the value of incorporating PET-CT in making this decision, observing that the most profitable strategy for managing the neck after treatment with chemoradiotherapy is to reserve the cervical dissection only for patients with residual disease shown on PET-CT.^17^

The ideal time to perform the first PET-CT after treatment is something that generates controversy, since it can affect its diagnostic precision.^5^ Many authors state that the precision of PET-CT increases as more time passes after the end of therapy, with better results when performed between 10 to 12 weeks after finishing treatment,^16^, ^18^ in order to avoid false positives,^19^, ^20^ since the error rate decreases over time.^21^ In cases where the PET-CT scan raise doubts, a repetition of the PET-CT can help guide the management of the patient. On the contrary, other authors recommend performing the first PET-CT post-treatment at 8 weeks.^22^

Regarding the detection of distant post-treatment metastases, we obtained a 0.913 correlation using only PET-CT. This fact, added to the fact that it is a complete body tracking technique, makes it more accurate than other methods of imaging. As in the detection of pathological adenopathies, they are the only two sections where there is greater precision with respect to untreated patients (V of Cramer 0.901).

When analysing the same stage parameters in treated patients, by using a single T, N and M diagnostic test, we observed a low correlation between the results based solely on CT with respect to the one based solely on PET-CT, which raises the question of which of the two tests is more accurate. When issuing the re-stage tumor, the correlation is 0.451. A figure too low in staging a tumor, coinciding only in 56.0% of the cases analysed. However, when comparing the correlation of both tests separately, we observed that PET-CT was more accurate than CT, since the correlation increases significantly when using only the first imaging test (Cramer’s V 0.858 vs. 0.565). The correlation in untreated patients between the two was notably higher (0.855), which indicates that the performance of both tests varies when used in previously treated patients.

In the case of re-staging of the primary tumor, we observed a compatibility of 0.397 between both imaging methods with a coincidence of 69.6% of the occasions, but when observing the individual analysis of each test, we observed a better correlation of PET-CT with respect to CT (Cramer’s V 0.852 vs. 0.502). The correlation is also lower than with respect to the comparison of both techniques of imaging in untreated patients (Cramer’s V 0.915),^7^ which indicates a greater precision when using PET-CT as the only image-based diagnostic method.

Similarly, when conducting a post-treatment study of the presence of lymph nodes in the neck, the correlation between both tests was 0.535, coinciding 79.2% of the cases studied. But that of the PET-CT with respect to the Tumor Board was higher than that of CT (Cramer’s V = 0.969 vs. 0.531). The same happened in the case of the detection of distant metastases, where the compatibility between each evidence was low at (0.579) and a coincidence of 88.8% of the cases analysed. However, PET-CT had a greater agreement with respect to the Tumor Committee than CT (Cramer’s V = 0.913 vs. 0.673).

## Conclusions

We consider it necessary to obtain post-treatment images to evaluate the need to perform salvage surgery in the event of a residual disease or recurrence, or the detection of new primary tumors, as this improves the diagnosis of the patient.

Residual neck disease after chemoradiotherapy was present in 30%–60% of patients. Even in patients with a complete clinical response, 16%–39% had microscopic tumor remains.^23^, ^24^, ^25^, ^26^ Therefore, physical examination and conventional imaging techniques were not always sufficient.^27^ In the past, it was common to perform routine cervical emptying after a concomitant chemoradiotherapy in patients with advanced stage tumors, although this procedure was associated with considerable mortality.^24^

Therefore, we have shown that in patients who have received treatment previously, regardless of the modality, there is a greater correlation in the restaging with respect to the use of PET-CT by the Tumor Board as the only Image-based diagnostic method. These findings are similar to those of other studies that have revealed that requesting a post-treatment PET-CT has had a significant impact on the management of the patient with a locally advanced CCC, especially to assess response to treatment and determine the need for neck dissection.^6^ That is why we consider that the realization of a post-treatment PET-CT is a fact to take into account to increase the precision in the therapeutic management of these patients and improve the prognosis of themselves.

## Conflicts of interest

The authors declare no conflicts of interest.
